# Graphene Oxide Reinforced Alginate/PVA Double Network Hydrogels for Efficient Dye Removal

**DOI:** 10.3390/polym10080835

**Published:** 2018-07-28

**Authors:** Cuiyun Liu, Hongyu Liu, Tianhui Xiong, Airong Xu, Bingli Pan, Keyong Tang

**Affiliations:** 1 Chemical Engineering and Pharmaceutics School, Henan University of Science and Technology, Luoyang 471023, China; liucy1218@126.com (C.L.); 18438608207@163.com (T.X.); airongxu@126.com (A.X.); blpan@haust.edu.cn (B.P.); 2 Material Science and Engineering School, Zhengzhou University, Zhengzhou 450001, China

**Keywords:** sodium alginate, PVA, graphene oxide, double network hydrogel, adsorption

## Abstract

Dually crosslinked graphene oxide reinforced alginate/polyvinyl alcohol (PVA) double network (DN) hydrogels were prepared via a facile freeze/thaw method followed by soaking in a Ca^2+^ solution. The morphology and structure of the hydrogels were systematically examined by Fourier transform infrared spectroscopy (FTIR), X-ray diffraction (XRD), scanning electron microscopy (SEM), and thermogravimetric analysis (TGA). The effects of pH, dosage of hydrogel, adsorption time, and temperature on the adsorptive property of DN hydrogels towards methylene blue (MB) were also studied. Results indicated that the hydrogels exhibited typical 3D porous structures and had an efficient adsorption effect towards MB due to strong interactions between DN hydrogels and MB molecules. The adsorption isotherm was found to coincide with the Langmuir model with a monolayer adsorption. The highest adsorption capacity of DN hydrogels for MB was examined as 480.76 mg·g^−1^.

## 1. Introduction

Water pollution originating from all kinds of organic dyes is on the rise due to the rapid development of printing, dyeing, and chemical industry. On account of containing strongly toxic, highly colored, and poorly biodegradable organic dyes, dyestuff wastewater affects the ecological environment and human health seriously [1,2,3]. Methylene blue (MB) is a water-soluble azo dye extensively used for dye printing, biological staining, as a chemical indicator, and so on. In order to reduce the pollution of dyes to water, contamination of MB for instance, many sewage treatment technologies, including biological treatment, chemical oxidation, catalytic ozonation, membrane separation, and adsorption have been developed so far [4,5,6]. Among them, adsorption is a simple, effective, low-cost, and highly available method. However, the exploitation of highly efficient, environmentally friendly adsorbents remains a challenge due to the complexity of wastewater.

In recent years, hydrogels with 3D structures as adsorptive materials for the purpose of dye wastewater treatment have drawn widespread attention [7,8,9]. Dye molecules can permeate into 3D hydrogels with high adsorption rates, integrating with molecular chains of hydrogels by forming hydrogen bonding or electrostatic interactions; therefore, these dye molecules can be readily immobilized by the hydrogel chains. Consequently, porous hydrogels exhibit high adsorption capacities towards different dyes via their versatile structures and properties. Sodium alginate (SA) is a biological linear natural polymer with the merits of biodegradability, low-cost, and non-toxic properties as well as a wealth of sources. It has been widely studied in wastewater treatment applications because of its preferable adsorption property [10,11]. However, the hydrogels obtained from SA exhibit drawbacks such as decreased strength, low superficial area, and weak chemical stability, therefore restricting its application in wastewater treatment. Double network (DN) hydrogels comprising two interpenetrating and cross-linked polymer networks have gained tremendous attention due to their enhanced mechanical property. Many investigations have reported that DN hydrogels exhibited improved mechanical properties and high adsorption capacities for heavy metals and dyes [12,13,14]. For example, a SA/polyvinyl alcohol (PVA) DN hydrogel was prepared for the purpose of achieving enhanced mechanical properties and electrical conductivity [15]. Moreover, the DN hydrogels can be further reinforced by incorporating inorganic materials, such as graphene sheets.

So far, graphene and its derivates have attracted enormous interest due to their unique properties, such as large special surface area, excellent mechanical properties, and unapproachable barrier properties [16,17,18,19]. Compared with hydrophobic graphene, graphene oxide (GO) sheets decorated with a mass of hydroxyl, carboxyl, epoxy, and carboxide groups exhibit good water solubility and benign compatibility with hydrogels [20]. For example, a mechanically robust and printable 3D hydrogel based on alginate and graphene oxide was reported by Liu et al. [21]. However, there have been few studies conducted to examine the influence of graphene sheets on DN hydrogels. Based on these considerations, we herein report a graphene oxide reinforced SA/PVA DN hydrogel for MB adsorption application via a facile freeze/thaw method followed by soaking in a Ca^2+^ solution. The construction of SA/PVA DN structures greatly increased the mechanical property of SA hydrogels. Meanwhile, the incorporation of graphene oxide sheets into the hydrogels not only further enhanced the mechanical property, but also affected the morphology of the DN hydrogels, yielding smaller pore size and larger pore density.

## 2. Experimental

### 2.1. Materials

Sodium alginate (*M*_w_ ~250,000 g·mol^−1^), polyvinyl alcohol (*M*_w_ ~74,800 g·mol^−1^), concentrated sulfuric acid (95%), hydrochloric acid, potassium permanganate, sodium nitrate, and hydrogen peroxide (30%) were purchased from Sinopharm Chemical Reagent Co. Ltd., Shanghai, China. Natural flake graphite (>99%) was bought from Nanjing Pionerr Nano Co. Ltd., Nanjing, China. Anhydrous calcium chloride was purchased from Tianjin Fengchuan Chemical Reagent Technologies Co. Ltd., Tianjin, China. All materials were directly used without further purification. 

### 2.2. Fabrication of Graphene Oxide (GO) Reinforced SA/PVA DN Hydrogels

Graphite oxide was prepared by a modified Hummer’s method [22]. The as-prepared brown graphite oxide was purified by washing with huge amounts of deionized (DI) water to remove residual acids. Then, graphite oxide sponge was obtained by freeze drying. Finally, 0.5 g graphite oxide sponge was ultrasonicated in 500 mL DI water for 30 min to get a brown GO solution. 

**Figure 1:**
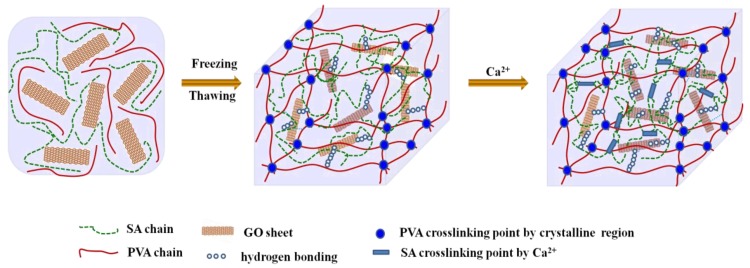
Schematic of crosslinking mechanism of graphene oxide (GO) reinforced double network (DN) hydrogel.

PVA solution was prepared by dissolving 5 g PVA powder in 100 mL DI water under continuous magnetic stirring at 90 °C. After natural cooling, 5 g SA powder was added into the PVA solution. After magnetic stirring for 2 h, the uniform mixture was degassed and then poured into a mould. Subsequently, the mixture was placed in a freezer and kept at −20 °C for 18 h. Then, the frozen hydrogels were allowed to thaw at room temperature (RT) until the ice melted. This freezing/thawing process was repeated 3 times to form physical crosslinked points between PVA chains. Finally, the hydrogel specimens were dipped into a 4 wt % CaCl_2_ (200 mL) solution for 6 h to crosslink SA chains, yielding SA/PVA DN hydrogels. Residual Ca^2+^ within the DN hydrogels was removed by swelling hydrogels in a mass of DI water. GO reinforced SA/PVA DN hydrogels were prepared by the same procedures with loadings of 100, 300, 500, and 700 mg GO, respectively. The as-prepared hydrogels with GO contents of 1, 3, 5, and 7 wt % were designated as GO1-SA/PVA, GO3-SA/PVA, GO5-SA/PVA, and GO7-SA/PVA, respectively. The preparation procedures and crosslinking mechanisms of the GO reinforced DN hydrogels are schematically represented in Figure 1. 

### 2.3. Characterizations

Fourier transform infrared (FTIR) measurement was carried out by using a potassium bromide micropellet technique on a Nicolet iS50 spectrometer (Thermo Fisher Scientific, Waltham, MA, USA). X-ray diffraction (XRD) analysis was performed on a D8 X-ray diffractometer (40 kV, 40 mA, *λ* = 0.154 nm, Bruker, Karlsruhe, Germany) by using a powder diffraction method. Thermogravimetric analysis was conducted by a TQ50 from room temperature to 700 °C at a heating rate of 10 °C·min^−1^ in nitrogen flow (TA Instruments, New Castle, DE, USA). Prior to scanning electron microscopy (SEM) study, the fractured freeze-dried hydrogels were coated with a thin layer of gold (JSM-5610LV, Japan Electronics Co. Ltd., Tokyo, Japan). The detection of MB concentration was performed using a UV–visible spectrophotometer (UV5200, Metash instrument Co. Ltd., Shanghai, China). Tensile test experiments were carried out on a CMT6104 electronic-universal testing machine (Shenzhen Skyan Power Equipment Co. Ltd., Shenzhen, China).

### 2.4. MB Adsorption Studies

MB adsorption experiment was performed by shaking a certain mass of GO reinforced DN hydrogels in 25 mL MB solution with an agitating speed of 100 rpm at 30 °C for 15 h to reach the absorbance equilibrium state. HCl and NaOH solutions were used in order to adjust the pH values of the adsorption solutions. Absorbance of the solution was measured by a UV–visible spectrophotometer under a wavelength of 664 nm. The influences of GO loadings (under pH 6.5) and pH on the adsorption were examined by soaking a 0.025 g sample into 25 mL of MB solution (200 mg·L^−1^) for 15 h. The influence of the initial concentration of MB on the adsorption was performed by adding 0.025 g of specimen into 25 mL of MB solution (50, 100, 150, 200, 300, 400, 500, 700, and 900 mg·L^−1^, respectively) for 15 h (pH = 6.5). The effect of contact time on the adsorption was investigated by soaking 0.15 g of sample into 150 mL of MB solution under pH of 6.5. The adsorption capacity (*q*_t_) and removal efficiency (*Re*) were calculated from the following expressions:

(1) $$q_{t}=\frac{\left(C_{0}{-C}_{t}\right)V}{m}$$

(2) $$Re(\%)=\frac{C_{0}{-C}_{t}}{C_{0}}\times 100\%$$

where *q*_t_ is the adsorption capacity (mg·g^−1^) and *C*_0_ is the initial MB concentration, whereas *C*_t_ is the MB concentration at any time, *t* (mg·L^−1^). *V* was the volume of the experimental solution (mL) and *m* was the mass of adsorbent (g). 

In order to investigate the adsorption process and adsorption mechanism of GO reinforced DN hydrogels, three kinds of adsorption kinetic equations, namely, the pseudo-first-order equation, pseudo-second-order equation, and intraparticle diffusion equation, were used to fit the experimental data [23]. 

The pseudo-first-order kinetic model is expressed as:

(3) $$\ln(q_{e}-q_{t})=\ln q_{e}-k_{1}t$$

where *q*_t_ and *q*_e_ stand for the adsorption capacities of DN hydrogels (mg·g^−1^) at *t* and the equilibrium state, respectively. *k*_1_ (min^−1^) is the rate constant.

The pseudo-second-order equation is expressed as:

(4) $$\frac{t}{q_{t}}=\frac{1}{k_{2}q_{e}^{2}}+\frac{t}{q_{e}}$$

where *k*_2_ (g·mg^−1^·min^−1^) is the pseudo-second-order adsorption rate constant. 

The intraparticle diffusion model is expressed as:

(5) $$q_{t}=k_{i}t^{\frac{1}{2}}+C$$

where *k*_i_ is the rate constant of the intraparticle diffusion model (mg∙g^−1^·min^−1/2^) and *C* is a constant standing for the boundary layer effects. 

## 3. Results and Discussion

### 3.1. Formation Mechanism of GO Reinforced SA/PVA DN Hydrogels

The dual crosslinking mechanisms of GO reinforced SA/PVA DN hydrogels were schematically presented in Figure 1. As could be seen, PVA chains were crosslinked by forming crystalline regions during the freezing/thawing treatments of PVA solution. On the other hand, alginate chains were crosslinked by Ca^2+^ ions, as described elsewhere [24]. Furthermore, the presence of GO offered hydrogen bonding interaction between GO sheets and PVA/SA chains. Therefore, GO sheets worked as additional crosslinking points in SA/PVA DN hydrogels. This conclusion was further confirmed by FTIR and SEM studies.

### 3.2. FTIR Analysis

**Figure 2:**
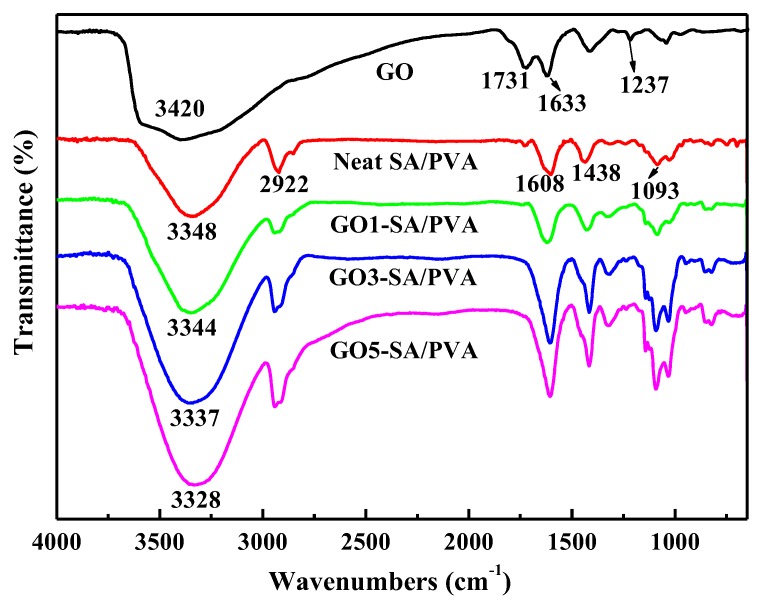
FTIR spectra of GO, DN hydrogel, and DN hydrogels with different GO loadings.

FTIR spectrometry was employed for monitoring the interactions between GO sheets and polymer networks. As shown in Figure 2, the FTIR spectrum of GO exhibited characteristic peaks of –OH, C=O, C=C, and C–O–C at 3420, 1731, 1633, and 1237 cm^−1^, respectively. For neat SA/PVA, the predominant peaks located at 3348 and 2922 are ascribed to the stretching vibrations of –OH and –CH_2_, respectively. The peaks at 1608 and 1438 cm^−1^ are attributed to the asymmetric and symmetric stretching vibrations of carboxylate ions, respectively [25]. The characteristic peak located at 1093 cm^−1^ was due to the stretching vibration of C–O–C. In comparison with neat SA/PVA, GO reinforced SA/PVA DN hydrogels exhibited lower wave numbers, possibly due to the dissociation of hydrogen bonding among the hydroxyl groups in PVA and formation of new hydrogen bonding between the hydroxyl groups of PVA/SA with hydroxyl groups of GO sheets [18]. 

### 3.3. Thermogravimetric Analysis

**Figure 3:**
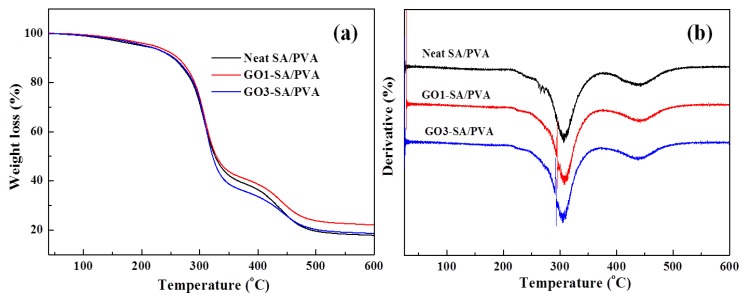
(**a**) TGA and (**b**) differential thermogravimetric analysis (DTG) curves of DN hydrogel and GO reinforced DN hydrogels composites.

TGA (TA Instruments, New Castle, DE, USA) and corresponding differential thermogravimetric analysis (DTG) curves are shown in Figure 3a,b, respectively. TGA thermograms exhibited the good thermal stability of specimens below 250 °C. This slight weight loss (8%) was attributed to the dissociation of water [19]. Sharp mass loss was observed around 265–340 °C due to the decomposition of polymer networks. It was noted that the weight loss of DN hydrogels with different GO loadings differed greatly. GO3-SA/PVA exhibited a weight loss of 52%, whereas GO1-SA/PVA exhibited a weight loss of 48%. However, the introduction of GO into SA/PVA DN hydrogels did not improve its thermal stability much.

### 3.4. XRD Analysis

**Figure 4:**
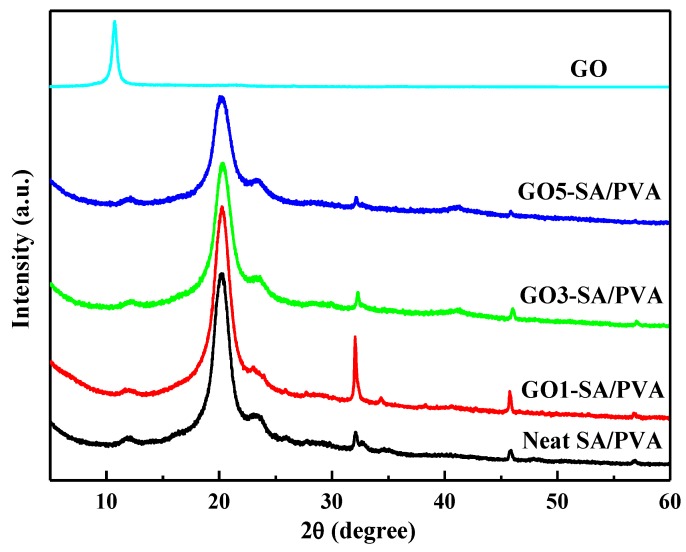
XRD patterns of GO, neat DN hydrogel, and GO reinforced DN hydrogels.

Figure 4 shows the XRD patterns of GO and GO reinforced SA/PVA specimens. As was shown, the XRD pattern of GO exhibited a sharp peak at 2θ = 19.5°. In the case of neat SA/PVA, the peak located at 2θ = 11.9° was due to the amorphous diffraction peak of SA. The peaks located at 2θ = 32°, 34°, and 45° were due to the presence of excrescent calcium ions in SA [26]. The sharp peak presented at 2θ = 20.2° was ascribed to the diffraction peak of PVA [18]. Interestingly, with increasing contents of GO, the intensity of the PVA peak increased slightly, followed by decreasing greatly. The changes in peak intensity demonstrated the corresponding changes in the degree of crystallinity of the SA/PVA hydrogels. It had been reported that the introduction of GO could affect the degree of crystallinity of PVA [18]. The largest degree of crystallinity of PVA in GO1-SA/PVA indicated that the PVA chains in GO1-SA/PVA exhibited the biggest physical crosslinking density by forming crystalline regions. Therefore, it was reasonable to observe the variation tendency in the XRD diffraction peaks of GO reinforced DN SA/PVA hydrogels. The reason was that the addition of nanofiller GO could impact on the crystallization behavior of crystalline polymers due to the heterogeneous nucleation of GO sheets. On the other hand, GO1-SA/PVA also exhibited the highest peaks at 2θ = 32° and 34°, in comparison to other diffraction peaks. This might be due to the relatively higher amount of residual calcium ions inside the GO1-SA/PVA. 

### 3.5. SEM Characterization

**Figure 5:**
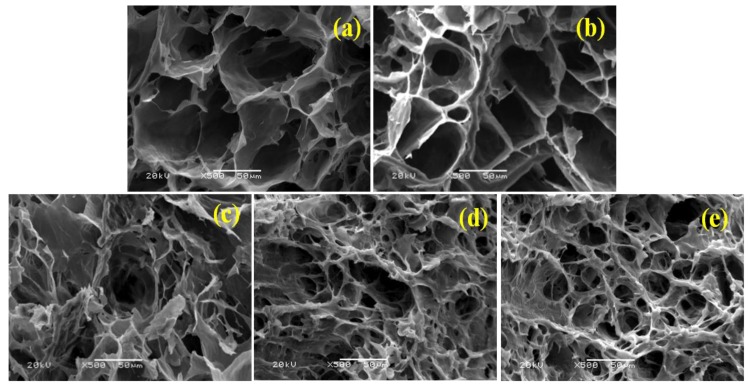
SEM images of SA/PVA with different GO loadings: (**a**) neat SA/PVA; (**b**) GO1-SA/PVA; (**c**) GO3-SA/PVA; (**d**) GO5-SA/PVA; (**e**) GO7-SA/PVA.

For further investigating the influence of GO sheets on the morphology of SA/PVA DN hydrogels, samples with different GO loadings were freeze-dried, then coated with a thin layer of gold and subjected to SEM examinations. The results were shown in Figure 5. It could be observed that all SA/PVA DN hydrogels exhibited a porous 3D structure, consistent with previous reports [27,28]. However, with increasing GO contents, the SA/PVA composites exhibited smaller pore size, demonstrating the formation of an improved degree of crosslinking. The crosslinking of SA/PVA by GO sheets via hydrogen bonding interactions accounted for this improved degree of crosslinking. Moreover, this increased degree of crosslinking could also enhance the mechanical property of SA/PVA DN hydrogels.

### 3.6. Mechanical Properties

**Figure 6:**
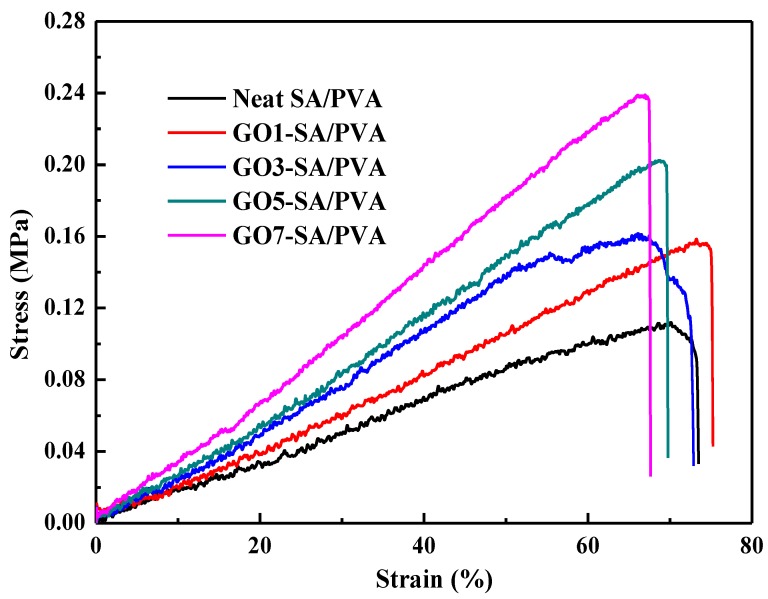
Stress-strain curves of neat DN hydrogel and GO reinforced DN hydrogels.

The stress-strain curves of the SA/PVA DN hydrogels with different GO loadings are shown in Figure 6. It is known that the degree of crystallinity, degree of crosslinking, and nanofiller dosage can influence the mechanical properties of composites greatly. As depicted, with increasing GO amounts, the SA/PVA DN hydrogels exhibited a tendency to decrease after an increase in degree of crystallinity. On the other hand, the degree of crosslinking was always increased with increasing GO contents. On account of the enhancements by GO sheets and degree of crosslinking, the fracture strength of GO reinforced SA/PVA hydrogels increased from 0.11 MPa for neat SA/PVA to 0.24 MPa for GO7-SA/PVA. In addition, it is worthy to note that the elongation at break of the composite hydrogels increased first, followed by decreasing. The increase in elongation at break for SA/PVA DN hydrogels with a small amount of GO can be ascribed to the formation of hydrogen bonding interaction between GO and polymer chains, as described by Fan et al. [29]. However, excessive embedment of GO sheets will restrict the elasticity of the polymer chains, therefore resulting in a reduction in elongation at break [30]. 

### 3.7. MB Adsorption

**Figure 7:**
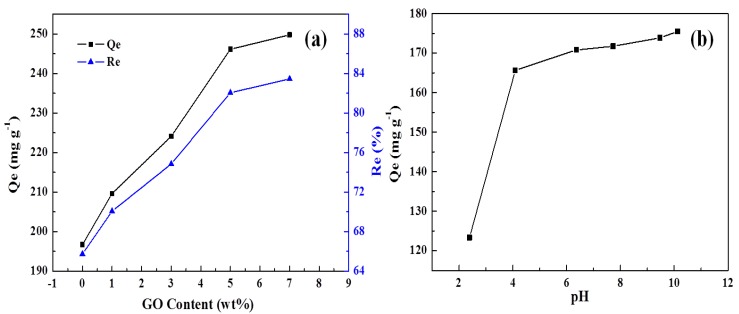
Effects of (**a**) GO content and (**b**) solution pH on the methylene blue (MB) adsorption.

As described previously in Figure 5, GO content affects the morphology of the GO reinforced DN hydrogels greatly. With loading more GO, the composite hydrogels exhibit smaller pore size and higher porosity, therefore affecting the adsorption behavior of MB. The influence of GO content on the adsorption of DN hydrogels for MB is shown in Figure 7a. It can be observed that increased GO content results in an improved adsorption capacity for MB. First, higher GO content results in higher porosity and larger specific area, which can obviously improve the adsorption capacity. Second, GO sheets were functionalized with a vast number of hydroxyl and carboxyl groups. These versatile groups can interact with MB molecules by electrostatic or hydrogen bonding interactions. Finally, GO and MB molecules can have strong π–π interactions. Based on these factors, improving GO content produces enhanced adsorption capacity. However, when the GO content is higher than 5%, the increase tendency in adsorption capacity is slowed down, probably due to the aggregation of GO sheets. 

pH is one of the important factors which influence the adsorption properties of DN hydrogel for MB. The effect of pH on the MB adsorption is shown in Figure 7b. As can be seen, with increasing the pH values of the solution from 2.4 to 6.4, the adsorption capacity increases greatly from 123.40 to 165.51 mg·g^−1^. On the other hand, when further increasing the pH values from 6.4 to 9.5, the adsorption capacity does not change much. Due to the presence of versatile oxygen-containing groups decorated in DN hydrogels, such as hydroxyl and carboxyl groups, MB can be adsorbed onto the DN hydrogels by either electrostatic interaction or hydrogen bonding interaction. Different pH values not only influence the charge distribution of DN hydrogels, but also the ionization of DN hydrogels and MB. Therefore, the adsorption behavior of DN hydrogels towards MB was greatly influenced by pH values. At low pH values, the carboxyl groups of SA and GO were protonated. Consequently, it reduces the electrostatic interactions between the DN hydrogels and MB molecules. In addition, excess proton ions will transform the hydroxyl groups of DN hydrogels into –OH_2_^+^ groups. Then, the electrostatic repulsion between –OH_2_^+^ groups and cationic dye MB further reduces the adsorption capacity [31,32]. Hence, at low pH values, the DN hydrogels exhibit extremely low adsorption capacities. When increasing pH values, the carboxyl groups of DN hydrogels were unprotonated as anions; the electrostatic interaction between the carboxylate radical and MB molecules accounts for the increased adsorption capacities for MB. The optimum pH was determined to be 6.4 for MB [33].

**Figure 8:**
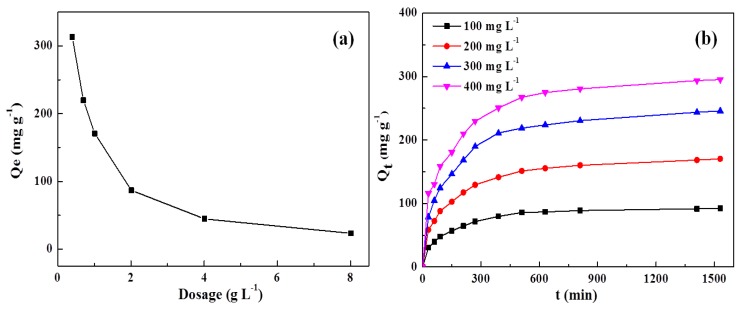
Effects of (**a**) dosage of adsorbent and (**b**) contact time on the MB adsorption.

The effect of DN hydrogel dosage on the adsorption was examined by soaking different amounts of hydrogels into 25 mL of MB solution with a concentration of 200 mg·L^−1^. As can be seen from Figure 8a, the equilibrium adsorption capacity of DN hydrogels for MB was decreased with increasing the dosage of DN hydrogels. When the dosages of DN hydrogel were changed from 0.5 to 8 g·L^−1^, the equilibrium adsorption capacities were reduced from 313.09 to 23.09 mg·g^−1^. For a certain amount of MB solution, DN hydrogels with lowered dosages would readily reach a saturation adsorption state due to the limited adsorption active points in DN hydrogels [34]. However, when increasing the dosage of DN hydrogels, the adsorption active points were in an unsaturated adsorption state, resulting in lowered adsorption capacities. The optimum adsorbent dosage was determined to be 1.00 g·L^−1^ for MB. 

The influence of contact time on the adsorption was shown in Figure 8b. As can be seen, DN hydrogels exhibit similar adsorption behavior for MB solutions with different concentrations. At the beginning of the adsorption, the adsorption capacity increases with increasing the contact time. This might be due to the large content of MB at the initial state, resulting in convenience for the MB to diffuse into the DN hydrogels. Upon further increasing contact time, the number of MB molecules in solution reduced and the active adsorption points in DN hydrogels were occupied by MB molecules, therefore resulting in a moderate increase in adsorption capacity [35].

The corresponding kinetic parameters and determination coefficients obtained from the slope and intercept according to these equations are shown in Table 1 and Table 2.

**Figure 9:**
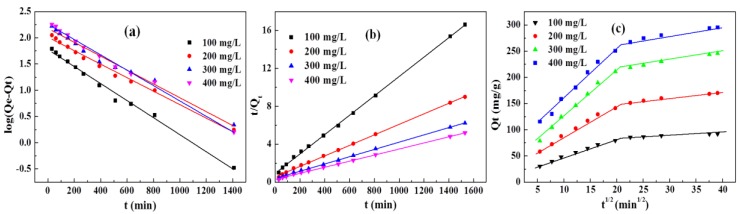
(**a**) Pseudo-first-order plots, (**b**) pseudo-second-order plots, and (**c**) intraparticle diffusion plots for MB adsorption.

In comparison with the pseudo-first-order model, the theoretical equilibrium adsorption capacities of the pseudo-second-order model shown in Figure 9b and Table 1 are closer to the experimental values. In addition, the correlation coefficients (*R*^2^) of the pseudo-second-order model are larger than that of the pseudo-first-order model, indicating that the pseudo-second-order model was applicable to the MB adsorption process [32]. Furthermore, the theoretical adsorption capacities of MB increase with increasing MB concentration, whereas the adsorption rate constant of the pseudo-second-order model decreases with increasing MB concentration due to the hindrance of higher concentrations of MB. After disclosing the adsorption process, the adsorption mechanism was further discussed by using the intraparticle diffusion model.

According to Figure 9c, all plots don’t pass through the origin, indicating that intraparticle diffusion was not the sole rate-controlling step. However, the majority of the correlation coefficients in Table 2 were larger than 0.98, demonstrating the adsorption mechanism can be explained by the intra-particle diffusion model. There are two stages in the adsorption curves of MB by DN hydrogels exhibiting different slopes. In the first stage, MB molecules diffuse from the solution into the surface of the DN hydrogels through large pores or channels inside the porous DN hydrogels. In the second stage, MB molecules diffuse into smaller pores and finally reach the equilibrium state [36]. 

Langmuir and Freundlich adsorption isothermal equations are widely used to describe the chemical adsorption behaviours [37]. Langmuir adsorption isotherm relies on the monolayer adsorption and the adsorption points being distributed uniformly among the surface of the adsorbent. The interaction between adsorbent and adsorbate is assumed to be strong enough. The Langmuir adsorption isotherm is expressed as:

(6) $$\frac{C_{e}}{q_{e}}=\frac{1}{q_{m}K_{L}}+\frac{C_{e}}{q_{m}}$$

where *q*_e_ and *c*_e_ are the equilibrium adsorption capacity (mg·g^−1^) and equilibrium concentration (mg·g^−1^), respectively. *q*_m_ is the maximum adsorption capacity (mg·g^−1^) and *K*_L_ is the rate constant of Langmuir adsorption isotherm. 

Freundlich adsorption isothermal is an empirical equation; normally used for elucidating a nonideal adsorption behaviour of adsorbent, it can be expressed as:

(7) $$\log qe=\log K_{F}+\frac{1}{n}\log C_{e}$$

where *K*_F_ is the rate constant of the Freundlich adsorption isotherm. 1/*n* is a constant related to adsorption capacity. According to Table 3, the 1/*n* value is 0.44, indicating that MB is easily adsorbed onto DN hydrogels by a Freundlich adsorption model. 

**Figure 10:**
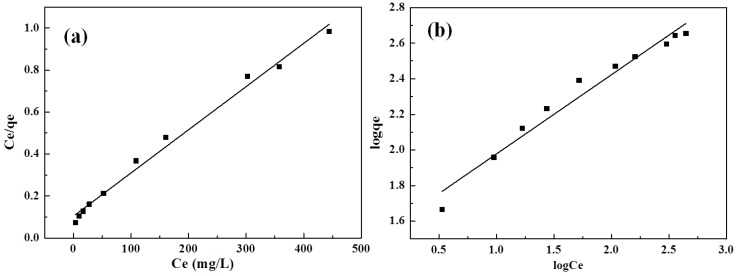
Langmuir (**a**) and Freundlich (**b**) adsorption isotherm models of MB.

The experimental data were fitted with the Langmuir and Freundlich adsorption isothermal equations, respectively, and the results are shown in Figure 10 and Table 3. The correlation coefficient (*R*^2^) of the Langmuir adsorption model is 0.9896, larger than that of the Freundlich adsorption model (0.9635), demonstrating that the Langmuir adsorption model is more in accord with the experimental data in comparison with the Freundlich adsorption model. 

In order to investigate the influence of temperature on the adsorption process, an adsorption experiment was performed at 298, 303, 313, and 323 K, respectively. Thermodynamic parameters, such as Δ*G*, Δ*H*, and Δ*S* were determined as:

(8) $$K_{c}=\frac{q_{e}}{C_{e}}$$

(9) $$\ln K_{c}=\frac{\Delta S}{R}-\frac{\Delta H}{RT}$$

(10) ΔG=ΔH−TΔS

where *T* (K) is temperature and Δ*H* (kJ∙mol^−1^), Δ*S* (J mol^−1^ K^−1^), and Δ*G* (kJ∙mol^−1^) are the changes in enthalpy, entropy, and Gibbs free energy, respectively. 

The results are given in Table 4. As is shown, all Δ*G* values are negative, indicating that the adsorption of MB is a spontaneous process. Furthermore, the value of Δ*G* increases with improving temperature, demonstrating that low temperature is favourable for MB adsorption. Δ*H* is negative, demonstrating that the adsorption is an exothermic process. Due to the adsorption of MB molecules onto the surface of DN hydrogels reducing the disorder, the values of Δ*S* also are negative. This is in line with a previous report [38].

## 4. Conclusions

In summary, dually crosslinked SA/PVA DN hydrogels reinforced by GO sheets were successfully prepared via a facile freeze/thaw process followed by soaking in a Ca^2+^ solution. The as-resulted hydrogels exhibited typical 3D porous structures. Contributed by the fascinating GO sheets, the porous hydrogels exhibited enhanced mechanical properties and excellent adsorption behaviors towards MB. Therefore, the GO reinforced SA/PVA DN hydrogels can be used as a nontoxic, biodegradable, low-cost dye adsorbent to treat dyestuff wastewater. The experimental adsorptive data coincided with the Langmuir model. The pseudo-second-order model could describe the adsorption behaviour of the DN hydrogel for MB well. Thermodynamic investigations elucidated that the adsorption of MB by DN hydrogels was a spontaneous and exothermic process.

## Figures and Tables

**Table 1:** Adsorption kinetic parameters of MB by adsorbents.

*C*_0_ (mg∙L^−1^)	Pseudo-first-order model				Pseudo-second-order model		
	*q*_e_ (exp)(mg∙g^−1^)	*k*_1_ (min^−1^)	*q*_e_ (cal)(mg∙g^−1^)	*R* ^2^	*k*_2_ (g∙mg^−1^∙min^−1^)	*q*_e_ (cal)(mg∙g^−1^)	*R* ^2^
100	92.01	0.0037	59.77	0.9912	1.14 × 10^−4^	97.66	0.9985
200	170.12	0.0029	102.78	0.9899	5.72 × 10^−5^	179.86	0.9981
300	245.75	0.0031	150.61	0.9845	3.93 × 10^−5^	260.42	0.9977
400	295.15	0.0034	181.75	0.9936	3.67 × 10^−5^	311.53	0.9982

**Table 2:** Parameters of intra-particle diffusion model for MB adsorption.

*C*_0_ (mg·L^−1^)	Step I			Step II		
	*K*_i1_ (mg·g^−1^·min^−1/2^)	*C* (mg·g^−1^)	*R* ^2^	*K*_i2_ (mg·g^−1^·min^−1/2^)	*C* (mg·g^−1^)	*R* ^2^
100	3.49	13.06	0.9899	0.39	77.04	0.9819
200	5.97	28.21	0.9868	1.09	127.98	0.9825
300	9.31	32.32	0.9924	1.61	183.07	0.9929
400	9.70	60.10	0.9867	1.61	233.05	0.9773

**Table 3:** Adsorption isotherm parameters of Langmuir and Freundlich models.

Langmuir model			Freundlich model		
*q*_m_ (mg·g^−1^)	*K*_L_ (L·mg^−1^)	*R* ^2^	*K*_F_	1/*n*	*R* ^2^
480.76	0.02	0.9896	34.11	0.44	0.9635

**Table 4:** Thermodynamic parameters for MB adsorption at different temperatures.

*T* (K)	*K*_c_ (L∙mg^−1^)	Δ*G* (kJ∙mol^−^^1^)	Δ*H* (kJ∙mol^−^^1^)	Δ*S* (J∙mol^−^^1^∙K^−^^1^)
298	6.94	−4.80	−15.45	−35.95
303	5.96	−4.49		
313	4.90	−4.14		
323	4.25	−3.89		
